# Inhibition of Geranylgeranyl Transferase-I Decreases Cell Viability of HTLV-1-Transformed Cells

**DOI:** 10.3390/v3101815

**Published:** 2011-10-10

**Authors:** Dustin C. Edwards, Katherine M. McKinnon, Claudio Fenizia, Kyung-Jin Jung, John N. Brady, Cynthia A. Pise-Masison

**Affiliations:** 1 Virus Tumor Biology Section, Laboratory of Cellular Oncology, Center for Cancer Research, National Cancer Institute, National Institutes of Health, Bethesda, MD 20892, USA; E-Mails: edwardsd2@mail.nih.gov (D.C.E.); jungk@kitox.re.kr (K.-J.J.); 2 Vaccine Branch, Center for Cancer Research, National Cancer Institute, National Institutes of Health, Bethesda, MD 20892, USA; E-Mails: mckinnonkm@mail.nih.gov (K.M.M.); feniziac@mail.nih.gov (C.F.)

**Keywords:** human T-cell leukemia virus type-1, HTLV-1, Tax, long terminal repeat, LTR, geranylgeranyltransferase, GGTI-298, small GTPase, NF-κB, p53, cell cycle

## Abstract

Human T-cell leukemia virus type-1 (HTLV-1) is the etiological agent of adult T-cell leukemia (ATL), an aggressive and highly chemoresistant malignancy. Rho family GTPases regulate multiple signaling pathways in tumorigenesis: cytoskeletal organization, transcription, cell cycle progression, and cell proliferation. Geranylgeranylation of Rho family GTPases is essential for cell membrane localization and activation of these proteins. It is currently unknown whether HTLV-1-transformed cells are preferentially sensitive to geranylgeranylation inhibitors, such as GGTI-298. In this report, we demonstrate that GGTI-298 decreased cell viability and induced G_2_/M phase accumulation of HTLV-1-transformed cells, independent of p53 reactivation. HTLV-1-LTR transcriptional activity was inhibited and Tax protein levels decreased following treatment with GGTI-298. Furthermore, GGTI-298 decreased activation of NF-κB, a downstream target of Rho family GTPases. These studies suggest that protein geranylgeranylation contributes to dysregulation of cell survival pathways in HTLV-1-transformed cells.

## 1. Introduction

An estimated 10–20 million people worldwide are infected with human T-cell leukemia virus type-1 (HTLV-1) [1], the etiological agent of adult T-cell leukemia (ATL) [2,3] and tropical spastic paraparesis/HTLV-1 associated myelopathy (TSP/HAM) [4–6]. ATL is characterized as a clonal lymphoproliferative disorder of CD4^+^ T-lymphocytes. Approximately 3 to 5% of HTLV-1-infected individuals develop ATL, which presents after a long period of clinical latency that can span several decades [7]. Disease onset is consistent with a multi-step process of T-lymphocyte immortalization and transformation. The mechanism by which infected individuals develop ATL is unknown, however the 40 kDa viral oncoprotein, Tax, has been associated with cellular transformation [8].

HTLV-1 Tax is essential for viral gene expression and Tax disrupts pathways involved in cell cycle regulation, apoptosis, and DNA damage responses through direct interaction with regulatory proteins and regulation of nuclear factor κB (NF-κB), cyclic AMP response element and activating transcription factor-1 (CRE/ATF-1), and serum response element (SRE) transcription pathways [8,9]. NF-κB transcriptional activation is triggered by phosphorylation of the upstream IκB kinase (IKK) complex through the phosphoinositide 3-OH kinase (PI3K)/Akt pathway [10]. Interestingly, phosphorylation of the NF-κB p65/RelA subunit at serine 536 contributes to the transcriptional inhibition of p53 in Tax-expressing cells. In HTLV-1-transformed cells, inhibition of p53 by Tax has a role in cell growth, cell cycle progression, and inhibition of apoptosis [11]. It has been shown that several chemical compounds induce apoptosis by reactivating p53 in HTLV-1-transformed cells [12–14]. Reactivation of p53, or induction of apoptosis in a p53-independent manner are therefore ideal strategies for the development of drugs to preferentially target ATL.

A new class of anticancer drugs has been recently demonstrated to decrease cell viability in multiple transformed cell lines through a p53-independent mechanism by inhibiting geranylgeranyl transferase 1 (GGTase-1) [15–17]. Geranylgeranyl transferase inhibitors (GGTIs), such as GGTI-298, inhibit a range of geranylgeranylated proteins, including the Rho family of small GTPases: Rho, Rac, and Cdc42 [18]. Activation of NF-κB by the Rho family has been well documented and GGTI-298 has been shown to affect the NF-κB pathway [17,19]. The effects of GGTI-298 are likely to be specific to cell type, external stimuli, and species [19,20]. In addition to regulation of NF-κB, Rho family members have been shown to regulate cellular gene transcription through CREB, SRF, and *c-Jun* transcription factor binding sites, each of which are present within Tax-responsive elements of the HTLV-1 promoter and have a role in viral gene expression [21–25]. Determination of whether HTLV-1-transformed cells are sensitive to geranylgeranyl transferase inhibitors would advance our understanding of how geranylgeranylated proteins regulate cell survival in these cells.

In this report we provide evidence that treatment of HTLV-1-transformed cells with GGTI-298 caused a significant decrease in cell viability. GGTI-298 induced G_2_/M phase accumulation and inhibited NF-κB in these cells. In contrast to other small molecule inhibitors, GGTI-298-mediated inhibition of NF-κB did not reactivate p53. GGTI-298 decreased Tax expression and transcriptional activation of the HTLV-1-LTR. Moreover, the decreased phosphorylation of IκB in GGTI-298-treated HTLV-1-transformed cells did not correlate with Tax protein levels. These studies suggest that protein geranylgeranylation contributes to dysregulation of cell survival pathways in HTLV-1-transformed cells.

## 2. Results and Discussion

### 2.1. GGTI-298 Decreases the Viability of HTLV-1-Transformed Cells

**Figure 1.**
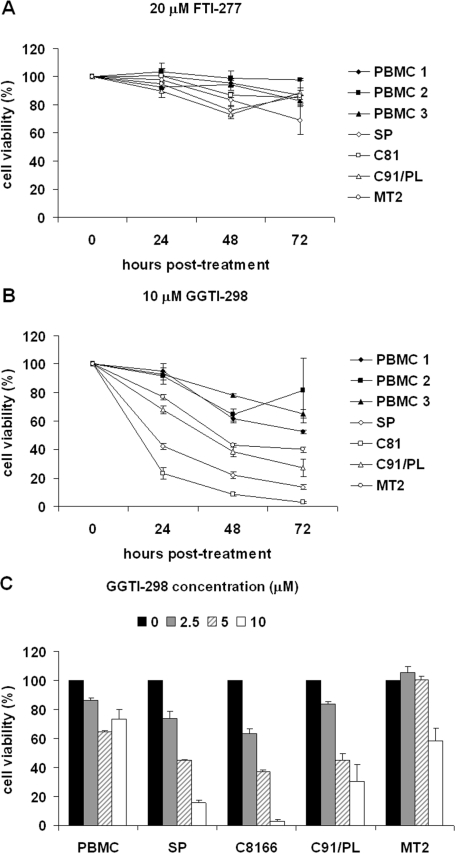
GGTI-298 decreases cell viability of Human T-cell leukemia virus type-1 (HTLV-1)-transformed cells. Control PBMCs from three donors and HTLV-1-transformed SP, C8166, C91/PL, and MT2 cells were treated with (**A**) 20 μM FTI-277 or (**B**) 10 μM GGTI-298. At the indicated times, cells were collected and cell viability determined. Values for cells treated with FTI-277 or GGTI-298 were compared to the signal for cells treated with DMSO solvent alone to determine percent cell viability. Error bars represent the standard error of the mean from three independent experiments performed in triplicate. (**C**) Control PBMCs and HTLV-1-transformed SP, C8166, C91/PL, and MT2 cells were treated for 72 hours with increasing amounts of GGTI-298 and cell viability measured as before. Error bars represent the standard error of the mean from three independent experiments performed in triplicate. For all GGTI298-treated cells p < 0.05 compared with mock-treated control, except MT2 cells treated with 2.5 and 5 μM GGTI-298.

We first sought to determine whether inhibitors of farnesyl transferase (FTase) or geranylgeranyl transferase I (GGTase I), catalysts of small GTPase prenylation, affect the viability of HTLV-1-transformed cells. Membrane association is critical for small GTPase activation and is mediated by covalent addition of C_15_ farnesyl or C_20_ geranylgeranyl isoprenyl groups to cysteine residues in the CAAX tetrapeptide motif near or at their carboxyl terminus [26]. Ras GTPases are modified by farnesylation while the majority of Rho family GTPases are geranylgeranylated. To examine sensitivity to prenylation inhibitors, HTLV-1-transformed cell lines C8166, C91/PL, and MT2 and control PBMCs from three different donors were treated with DMSO solvent, 20μM FTI-277 or 10 μM GGTI-298 (concentrations routinely used for other cancer types). As PBMCs were cultured in the presence of interleukin-2 (IL-2), HTLV-1-infected SP cells cultured in the presence of IL-2 served as an additional control to determine whether IL-2 contributed to the survival of drug-treated cells. Cells were harvested at 0, 24, 48, and 72 hours post-treatment. Cell viability was measured by a luminescent cell viability assay, which measures the ATP generated in viable cells. We found no significant difference in the viability of control PBMCs and HTLV-1-transformed cells treated with FTI-277 (Figure 1A). In contrast, the results presented in Figure 1B demonstrate that compared to control PBMCs, the HTLV-1-transformed cell lines were more sensitive to treatment with GGTI-298 than were control PBMCs (Figure 1B). The viability of C8166 cells was also analyzed by trypan blue exclusion assays and we found decreased cell viability, similar to that seen using the luminescent assay (data not shown). The sensitivity to GGTI-298 varied in the HTLV-1-transformed cells with C8166 cells being the most sensitive, followed by SP, C91/PL, and MT2 cells.

To determine the effects of increasing GGTI-298 concentrations on cell viability, final concentrations of 0, 2.5, 5, and 10 μM of the drug were added to the culture media of HTLV-1-transformed, HTLV-1-infected cells, and PBMCs for 72 hours. Following treatment, cell viability was determined. As shown in Figure 1C, the HTLV-1-transformed and infected cells were more sensitive to GGTI-298 and showed a dose-dependent loss of viability. After 72 hours of treatment with 10 μM GGTI, control PBMCs showed less than 30% decrease in viability. In contrast, HTLV-1-transformed cells, C8166 and C91/PL, and HTLV-1-infected SP cells showed a 70 to 95% reduction in viability. As above, MT2 cells were the least sensitive of the HTLV-1-transformed cells, with a 40% decrease in viability.

### 2.2. GGTI-298 Induces G_2_/M Phase Accumulation in HTLV-1-Transformed Cells

**Figure 2.**
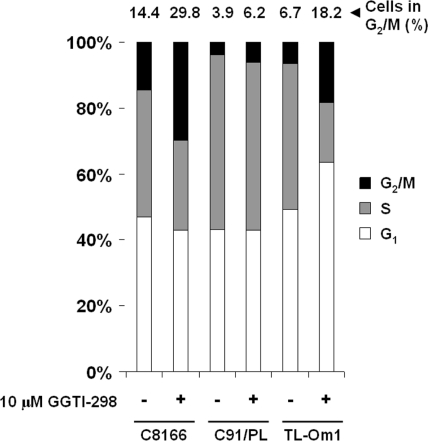
GGTI-298 induces G_2_/M phase accumulation in HTLV-1-transformed cells. HTLV-1-transformed C8166, C91/PL, and TL-Om1 cells were treated with DMSO or 10 μM GGTI-298. At 48 hours post-treatment, cells were fixed and stained with propidium iodide. Representative graphs from three independent experiments are shown for untreated and GGTI-298-treated C8166, C91/PL, and TL-Om1 cells. Percentage of cells in G_2_/M phase is shown above each bar.

GGTI-298 has been shown to inhibit Rho family GTPase activation and reduce cell viability in a number of cancer cell types, including transformed uninfected T-cells [17,27–35]. Although PBMCs have a reduced proliferation compared to HTLV-1-transformed cells, GGTI-298 clearly decreased the viability of HTLV-1-transformed cells. Therefore, we examined its effect on cell cycle progression in HTLV-1-transformed C8166 and C91/PL cell lines and a Tax-negative cell line, TL-Om1. Cells were treated with DMSO or 10 μM GGTI-298 for 48 hours and DNA content was analyzed by flow cytometry after staining with propidium iodide. As shown in Figure 2, GGTI-298 caused accumulation of C8166 and TL-Om1 cells in the G_2_/M phase. GGTI-298-treated C91/PL cells showed a small increase in G_2_/M phase accumulation in C9/PL cell, indicating that the effect of GGTI-298 on cell cycle distribution in HTLV-1-transformed cells is cell line dependent. Though both C8166 and TL-Om1 cells showed a two-fold increase in the percentage of cells in G_2_/M phase, only TL-Om1 cells showed an increase in cells in G_1_ phase following treatment with GGTI-298. In each of the cell lines treated with GGTI-298, there was a decrease in the percentage of cells in S-phase of the cell cycle. These results demonstrate that GGTI-298 affects the cell cycle checkpoint pathways by inducing accumulation of HTLV-1-transformed cells in the G_2_/M phase of the cell cycle. This is consistent with other studies that show check point effects in other tumor cell lines [15,29,32,36,37].

### 2.3. GGTI-298 Inactivates the NF-κB Pathway

**Figure 3.**
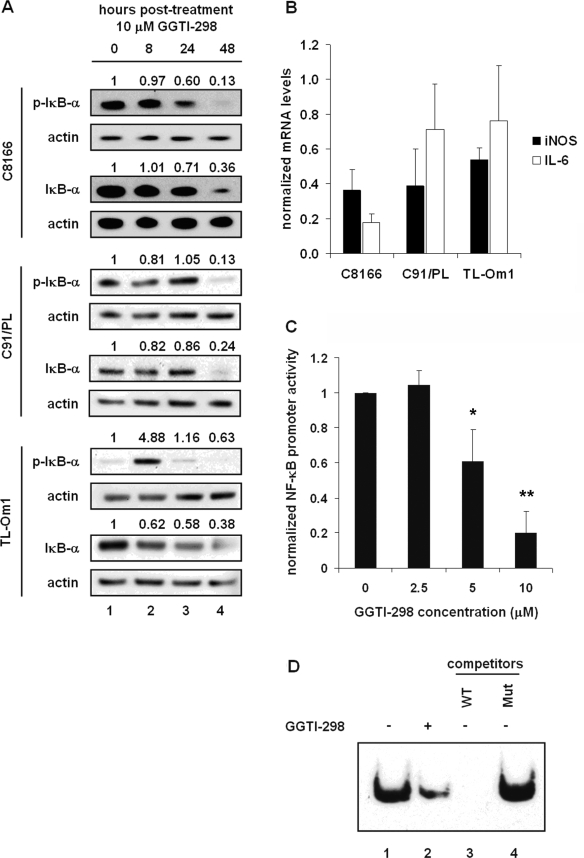
GGTI-298 inactivates the NF-κB pathway. (**A**) C8166, C91/PL, and TL-Om1 cells were treated with 10 μM GGTI-298 and whole cell extracts were prepared at 0, 8, 24, and 48 hours post-treatment. Immunoblots using antibodies to IκB, p-IκB or actin as indicated. Normalized densitometric volume is shown above each immunoblot set. (**B**) C8166, C91/PL, and TL-Om1 cells were treated with DMSO or 10 μM GGTI-298 and total RNA was extracted and retrotranscribed at 48 hours post-treatment. cDNA quantification for *18s* and NF-κB targets *iNOS* and *IL-6* was performed by real-time PCR. mRNA levels were normalized to *18s* RNA levels from the same well. Normalized mRNA levels from cells treated with DMSO solvent alone was set to 1 and mRNA levels from drug-treated cells were determined relative to this value. Error bars represent the standard error of the mean from three independent experiments performed in triplicate. (**C**) The 4×-NF-κB-Luc luciferase reporter plasmid was cotransfected with RSV-β-galactosidase reporter plasmid into C8166 cells. Four hours after transfection, cells were treated with increasing amounts of GGTI-298. Forty-eight hours after treatment with GGTI-298, cells were harvested and luciferase activities measured. Luciferase activity was normalized to β-galactosidase activity from the same well. Error bars represent the standard error of the mean from four independent experiments performed in duplicate. * *p* < 0.05; ** *p* < 0.005 compared with mock-treated control. (**D**) Effect of GGTI-298 on NF-κB DNA binding activity was assessed by EMSA using a consensus NF-κB oligonucleotide probe. C8166 cells were treated with 10 μM GGTI-298 and nuclear extracts were prepared 48 hours post-treatment. For the competition assay, 200-fold molar excess of unlabeled consensus or mutated oligonucleotide was added to the reaction.

GGTI-298 has been shown to inactivate AKT and the downstream NF-κB pathway [17,19]. Constitutive activation of NF-κB in HTLV-1-transformed cells plays a key role in cell survival, cell cycle progression, and prevention of apoptosis in HTLV-1-infected cells. To determine whether GGTI-298 affected NF-κB activation in HTLV-1-transformed cells, C8166, C91/PL, and TL-Om1 cells were treated with 10 μM GGTI-298 and collected at 0, 8, 24, and 48 hours post-treatment for immunoblot analysis. Decreased levels of IκBα, the inhibitor of NF-κB were seen by 48 hours post-treatment in C8166, C91/PL, and TL-Om1 cells (Figure 3A). Likewise, phosphorylated IκB (p-IκBα) levels decreased similarly to overall IκB levels. Interestingly, p-IκBα levels were initially low in TL-Om1 cells, peaked at 8 hours post-treatment, and similar to C8166 and C91/PL cells, decreased by 48 hours post drug treatment (Figure 3A).

To determine whether GGTI-298 affected NF-κB transcriptional activity, real-time PCR analysis was performed on downstream targets of NF-κB. Cells were treated for 48 hours with 10 μM GGTI-298 and total RNA was extracted and retrotranscribed. mRNA levels of the NF-κB-regulated genes *iNOS* and *IL-6* were normalized to *18s* RNA levels. A reduction in the levels of both NF-κB-regulated genes was seen in all three cell lines after treatment with GGTI-298. For C8166 cells, there was a decrease of 65 to 80% in *iNOS* and *IL-6* mRNA levels following drug treatment. While treatment of C91/PL and TL-Om1 cells with GGTI-298 showed a decrease of 60% and 40% in *iNOS* mRNA levels, *IL-6* mRNA levels were less affected with a decrease of about 25%. These data suggest that the mechanism of reduction in NF-κB-regulated gene expression by GGTI-298 is likely gene and cell-specific.

The effect of GGTI-298 on NF-κB transcriptional activity was directly analyzed using a 4×NF-κB-Luc luciferase reporter construct. Four hours post-transfection, C8166 cells were treated with increasing amounts of GGTI-298 or DMSO solvent. Forty-eight hours post-treatment, cells were collected and luminescence was measured. Cells treated with 5 μM GGTI-298 showed 40% less NF-κB transcriptional activity than cells treated with DMSO alone (Figure 3C). Treatment with 10 μM GGTI-298 reduced NF-κB transcriptional activity by 80%. These results suggest that GGTI-298 inhibits NF-κB transcriptional activity in HTLV-1-infected cells.

To further define the inhibition of NF-κB activity by GGTI-298, nuclear extracts from C8166 cells treated for 48 hours with 10 μM GGTI-298 were incubated with biotin-labeled NF-κB consensus sequence oligonucleotide probe and analyzed by electrophoretic mobility shift assay (EMSA). Consistent with the immunoblot and promoter activation results, GGTI-298 reduced NF-κB complex formation on its cognate DNA binding site (Figure 3D, lanes 1–2). The specificity of complex formation was demonstrated by competition assays in which excess unlabeled wild-type, but not mutant, NF-κB consensus oligonucleotide was able to compete for NF-κB complex formation (Figure 3D, lanes 3–4). Taken together, these results demonstrate that inhibition of protein geranylgeranylation inhibits the NF-κB pathway in HTLV-1-transformed cells.

### 2.4. The Effects of GGTI-298 on HTLV-1-Transformed Cells Are p53-Independent

Previous studies have demonstrated that Tax-mediated activation of a novel NF-κB pathway functionally inhibits p53 in HTLV-1-transformed cells [10]. NF-κB and AKT inhibitors or IκB mutants that inhibit NF-κB activation have been reported to reactivate p53 function in Tax-expressing cells. As GGTI-298 inhibited NF-κB in HTLV-1-transformed cells, we asked whether GGTI-298-induced G_2_/M phase accumulation and reduced viability was dependent on p53 reactivation.

**Figure 4.**
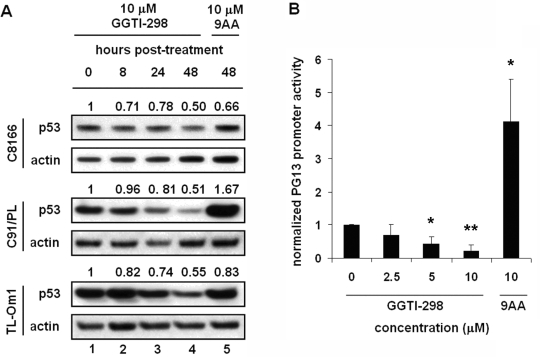

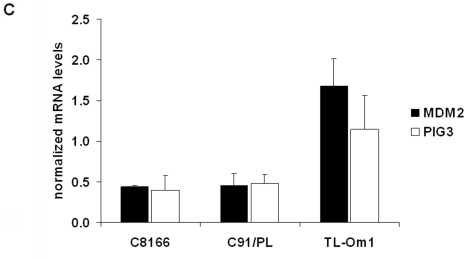
The effects of GGTI-298 on HTLV-1-transformed cells are p53-independent. (**A**) C8166, C91/PL, and TL-Om1 cells were treated with 10 μM GGTI-298 or 9AA and whole cell extracts were prepared at 0, 8, 24, and 48 hours post-treatment. Immunoblots using antibodies to p53 or actin as indicated. Normalized densitometric volume is shown above each immunoblot set. (**B**) PG13-Luc luciferase reporter plasmid was cotransfected with RSV-β-galactosidase reporter plasmid into C8166 cells. Four hours after transfection, cells were treated with increasing amounts of GGTI-298 or 10 μM 9AA. Forty-eight hours after treatment with GGTI-298, cells were harvested and luciferase activities measured. Luciferase activity was normalized to β-galactosidase activity from the same well. Error bars represent the standard error of the mean from three independent experiments performed in duplicate. * *p* < 0.05; ** *p* < 0.005 compared with mock-treated control. (**C**) C8166, C91/PL, and TL-Om1 cells were treated with DMSO or 10 μM GGTI-298 and total RNA was extracted and retrotranscribed at 48 hours post-treatment. cDNA quantification for *18s* and p53 targets *MDM2* and *IL-6* was performed by real-time PCR. mRNA levels were normalized to *18s* RNA levels from the same well. Normalized mRNA levels from cells treated with DMSO solvent alone was set to 1 and mRNA levels from drug-treated cells were determined relative to this value. Error bars represent the standard error of the mean from three independent experiments performed in triplicate.

To determine whether GGTI-298 affected p53 protein levels, C8166, C91/PL, and TL-Om1 cells were treated with 10 μM GGTI or 9AA and whole cell extracts collected at 0, 8, 24, and 48 hours post-treatment for immunoblot analyses. 9AA was included as a control since it has previously been shown to reactivate p53 function in HTLV-1-transformed cells [14]. We found no increase in p53 protein levels in GGTI-298-treated cells (Figure 4A). Note that the TL-Om1 cell line has a mutant functionally inactive p53 protein and thus we would not expect p53 to play a role in drug sensitivity in this cell line.

To directly analyze the effect of GGTI-298 on p53 transcriptional activity, we transfected C8166 cells with a p53-responsive reporter, PG13-Luc, that contains 13 repeats of the p53 response sequence upstream of the basal promoter driving luciferase. Four hours post-transfection, cells were treated with increasing amounts of GGTI-298, DMSO solvent alone or 9AA. Forty-eight hours post-treatment, cells were collected and luminescence measured. We observed a 4-fold increase in p53 transcriptional activity in cells treated with 9AA compared to cells treated with DMSO solvent alone (Figure 4B). In contrast, p53 transcriptional activity was not increased in cells treated with GGTI-298 at 2.5 to 10 μM concentrations, but rather the activity was decreased. This decrease in p53 transcriptional activity is consistent with the decrease in p53 protein levels (Figure 4A).

Real-time PCR analysis was performed on downstream targets of p53 in cells treated for 48 hours with 10 μM GGTI-298 (Figure 4C). mRNA levels of the p53-regulated genes *MDM2* and *PIG3* were normalized to *18s* RNA levels. In TL-Om1 cells, which contain a mutant p53, *MDM2* and *PIG3* mRNA levels increased 65% and 15%. In contrast, *MDM2* and *PIG3* mRNA levels decreased 50 to 60% in GGTI-298-treated C8166 and C91/PL cells. These results demonstrate that GGTI-298 does not reactivate p53 in HTLV-1-transformed cells and that the effects of GGTI-298 are p53-independent.

### 2.5. GGTI-298 Inhibits Transcription of the HTLV-1-LTR and Decreases Tax Expression

**Figure 5.**
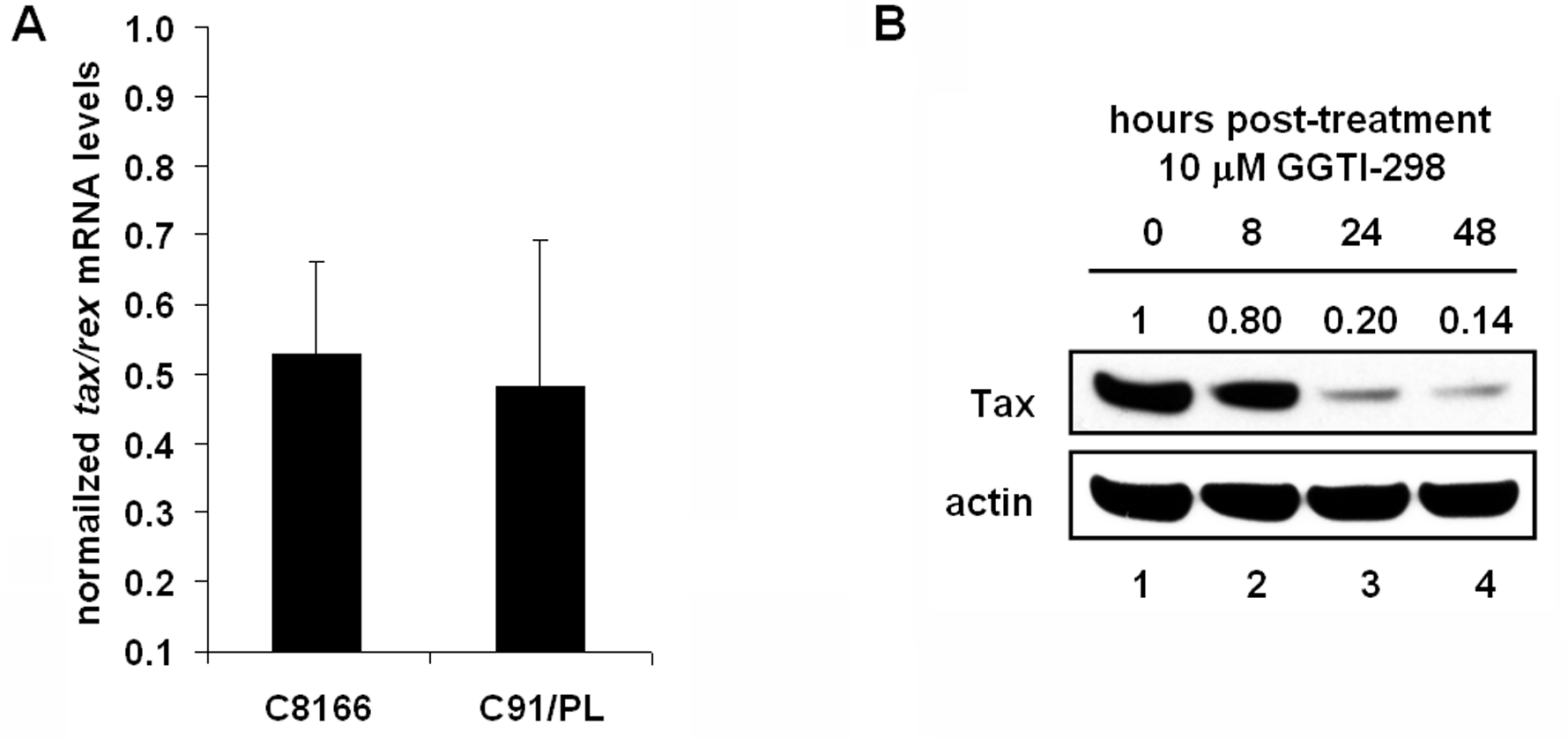

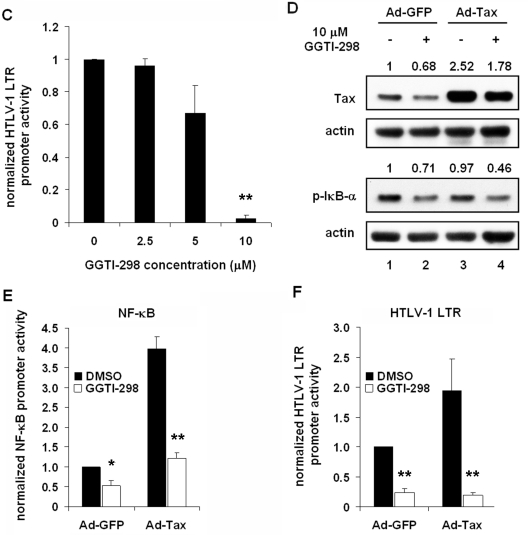
GGTI-298 inhibits transcription of the HTLV-1-LTR and decreases Tax expression. (**A**) C8166 and C91/PL cells were treated with DMSO or 10 μM GGTI-298 and total RNA was extracted and retrotranscribed at 48 hours post-treatment. cDNA quantification for *tax/rex* and *18s* was performed by real-time PCR. mRNA levels were normalized to *18s* RNA levels from the same well. Normalized mRNA levels from cells treated with DMSO solvent alone was set to 1 and mRNA levels from drug-treated cells were determined relative to this value. Error bars represent the standard error of the mean from three independent experiments performed in triplicate. (**B**) C8166 cells were treated with 10 μM GGTI-298 and whole cell extracts were prepared at 0, 8, 24, and 48 hours post-treatment. Immunoblots using antibodies to Tax or actin as indicated. Normalized densitometric volume is shown at top. (**C**) HTLV-1-LTR-Luc luciferase reporter plasmid was cotransfected with RSV-β-galactosidase reporter plasmid into C8166 cells. Four hours after transfection, cells were treated with increasing amounts of GGTI-298. Forty-eight hours after treatment with GGTI-298, cells were harvested and luciferase activities measured. Luciferase activity was normalized to β-galactosidase activity from the same well. Error bars represent the standard error of the mean from three independent experiments performed in duplicate. ** *p* < 0.005 compared with mock-treated control. (**D**) C8166 cells were infected with adenovirus expressing either GFP (Ad-GFP) or Tax (Ad-Tax). At 24 hours post-infection, cells were treated with 10 μM GGTI-298. At 48 hours post-infection, whole cell extracts were prepared and immunoblots were performed using antibodies to Tax, p-IκB, or actin as indicated. Normalized densitometric volume is shown above each immunoblot set. (**E**) The 4×-NF-κB-Luc or (**F**) HTLV-1-LTR-Luc luciferase reporter plasmid was cotransfected with RSV-β-galactosidase reporter plasmid into C8166 cells. Twenty-four hours post-transfection, cells were infected with Ad-GFP or Ad-Tax and treated 4 hours later with 10 μM GGTI-298. Forty-eight hours after treatment with GGTI-298, cells were harvested and luciferase activities measured. Luciferase activity was normalized to β-galactosidase activity from the same well. Error bars represent the standard error of the mean from three independent experiments performed in duplicate. * *p* < 0.05; ** *p* < 0.005 compared with mock-treated control.

In addition to regulation of NF-κB, Rho family members have been shown to regulate transcription of cellular genes through CREB, SRF, and *c-Jun* transcription factor binding sites, each of which are present in the Tax-responsive elements of the HTLV-1 promoter and have a role in viral gene expression [21–25]. Thus we next examined whether GGTI-298 affected transcriptional activation of the viral promoter. Real-time PCR analysis was performed on *tax/rex* mRNA levels in C8166 and C91/PL cells treated for 48 hours with 10 μM GGTI-298 (Figure 5A). As before, target mRNA levels were normalized to *18s* RNA levels. We found a 50% decrease in *tax/rex* mRNA levels in both C8166 and C91/PL cells following drug treatment. To determine whether GGTI-298 affects Tax protein expression, C8166 cells were collected for immunoblot analysis at 0, 8, 24, and 48 hours after treatment with GGTI-298. We found that Tax protein levels decreased approximately 75% after 24 hour treatment with GGTI-298 (Figure 5B). C8166 cells were treated with increasing amounts of GGTI-298 four hours after being transfected with an HTLV-1-LTR-luciferase construct. Cells were collected 48 hours post-treatment and luminescence was measured. We found a dose-dependent repression of viral promoter activity by GGTI-298 (Figure 5C). C8166 cells treated with 10 μM GGTI-298 showed 95% less HTLV-1 promoter activity as compared to cells treated with DMSO solvent alone. The decrease in HTLV-1-LTR activity in GGTI-298-treated C8166 cells may be in part due to diminishment of CRE pathway activity. In a CRE-luciferase reporter assay, we measured a 50% decrease in luciferase levels in C8166 cells treated with 10 μM GGTI-298 for 48 hours (data not shown). Together, this data shows that GGTI-298 significantly inhibits transcription of the HTLV-1-LTR and decreases Tax expression.

Thus, deactivation of NF-κB and accumulation of cells in G_2_/M phase in GGTI-298-treated HTLV-1-transformed cells may be, in part, due to decreased Tax expression and activity following inhibition of transcription pathways by GGTI-298. Indeed, preliminary experiments from another lab revealed that an HTLV-1-infected human T-cell line could not survive knockdown of Tax protein levels after being infected with a lentivirus that encoded siRNA targeting Tax [38].

We next determined whether the effect of GGTI-298 on the NF-κB pathway was due to decreased Tax protein expression in Tax positive cells [28]. C8166 cells were infected with either an adenovirus construct that expresses Tax (Ad-Tax) or GFP (Ad-GFP). Twenty-four hours post-infection, cells were treated with 10 μM GGTI-298 and incubated an additional 24 hours. Immunoblots were then performed and phosphorylation status of IκB analyzed (Figure 5D). As expected from previous experiments (Figure 3A), GGTI-298 decreased the levels of phosphorylated IκB in cells infected with Ad-GFP and Ad-Tax as compared to control treated cells. Interestingly, GGTI-298 reduced Tax protein levels in cells infected with the Ad-Tax construct (Figure 5D, lanes 3–4). Importantly, however, Tax protein expression was still significantly higher than in mock-treated cells infected with the Ad-GFP construct (Figure 5D, lane 1). Despite the high level of Tax expression, p-IκB levels still decreased in response to GGTI-298 treatment (Figure 5D). In addition, increased Tax expression did not abrogate the effects of GGTI-298 on NF-κB (Figure 5E) or HTLV-1-LTR (Figure 5F) transcriptional activity. In mock-treated cells expressing Ad-Tax, there was a four-fold increase in NF-κB transcriptional activity and a two-fold increase in HTLV-1-LTR activation compared to cells expressing Ad-GFP. Treatment of Ad-Tax and Ad-GFP expressing cells with GGTI-298 significantly inhibited NF-κB transcriptional activity and HTLV-1-LTR activation. While Ad-Tax activity was able to augment basal Tax-mediated transcriptional transactivation, it was unable to counter the effects of GGTI-298 on transcriptional repression. Together these results indicate that GGTI-298 decreases Tax protein levels and HTLV-1-LTR activity and that deactivation of the NF-κB pathway by the inhibitor does not correlate with Tax expression.

### 2.6. Discussion

Rho family GTPases regulate multiple signaling pathways that control cytoskeletal organization, transcription, cell cycle progression and cell proliferation. Overexpression of multiple Rho family members in human tumors suggests that these proteins are important in tumorigenesis and therefore potential candidates for therapeutic intervention [39]. Currently, a role for Rho family GTPases in the development of ATL is emerging. First, Tax has been reported to form complexes with several members of the Rho family including RhoA, Rac, Gap1m and Cdc42 [40]. Second, microarray analysis showed altered gene expression of numerous Rho GTPase and GTPase-binding proteins in ovine B-cells expressing bovine leukemia virus Tax (Tax_BLV_) [41]. Third, bisphosphonate incardonate, a compound that disrupts protein farnesylation and geranylgeranylation, has recently been demonstrated to induce apoptosis in HTLV-1-transformed cells [42]. Fourth, Nonaka *et al.* reported that inhibition of geranylgeranylation, but not farnesylation, induced apoptosis in HTLV-1-infected ATL cell lines [28]. Nonaka *et al.* further reported that the Rho family GTPases Rab5B and Rac1A were found to be prenylated in these cells. However, the authors did not define the mechanism used by GGTI-298 to cause cell death in the HTLV-1-transformed cells.

Here, we show that the prenylation inhibitor GGTI-298 decreased cell viability and induced G_2_/M phase accumulation in HTLV-1-transformed cells in a p53-independent manner. In contrast, the prenylation inhibitor FTI-277 did not affect cell viability in these cells. Treatment of HTLV-1-transformed cells with GGTI-298 decreased activation of the NF-κB signaling pathway. In addition, HTLV-1-LTR transcriptional activity was inhibited and Tax protein levels decreased following treatment with GGTI-298. The results of these experiments suggest a decrease in cellular transcription whose pattern differs by gene and cell type. However, more pathways need to be examined to determine specificity. We hypothesize that activated Rho family GTPases have a role in several cell signaling pathways that are dysregulated in HTLV-1-transformed cells and that geranylgeranyl transferase inhibitors may provide a novel class of therapeutic agents for treatment of ATL.

GGTI-298 may be useful for the study of other functions of Rho GTPases in HTLV-1-infected cells such as cell adhesion, migration, and chemotaxis. ATL is characterized by infiltration of circulating ATL cells into a variety of tissues. Fresh ATL cells from patients express lymphocyte function-associated antigen 1 (LFA-1) [43]. LFA-1 mediates adhesion of circulating leukocytes to endothelial ligands and mediates transendothelial migration of ATL cells [44]. Recently, it has been shown that GGTI-298 is able to diminish LFA-1 expression in T-acute lymphoblastic leukemia cells by inhibiting the GTPase Rap1b [45]. Interestingly, HTLV-1 p8 increases clustering of LFA-1 on the cell surface to enhance cell-cell adhesion to promote viral spread [46,47]. By demonstrating here that HTLV-1-transformed cells are sensitive to GGTI-298, we speculate that this compound could disrupt HTLV-1 p8-mediated modulation of cell adhesion in these cells by inhibiting LFA-1 clustering. Continued study and characterization of geranylgeranylated proteins, such as Rho family GTPases, will increase our understanding of the mechanisms that regulate infection and transformation of HTLV-1-infected cells.

## 3. Experimental Section

### 3.1. Preparation of PBMCs

Heparinized human peripheral blood was obtained from healthy volunteers at the NIH clinical center. Peripheral blood mononuclear cells (PBMCs) were isolated from healthy donors using Ficoll-Paque Plus (GE Healthcare, Chalfont St. Giles, UK) according to the manufacturer’s instructions. Isolated lymphocytes were washed in PBS and activated in the presence of 2 μg/mL phytohemagglutinin-L (PHA-L) for 24 hours before being cultured in RPMI media supplemented with 20% FBS, 2 mM l-glutamine, penicillin/streptomycin and 50 U/mL interleukin-2 (IL-2).

### 3.2. Cell Lines and Drug Treatment

The HTLV-1-transformed cell lines C8166, C91/PL, and MT2, the ATL cell line TL-Om1 were maintained in RPMI media supplemented with 10% FBS, 2 mM L-glutamine and penicillin/streptomycin. IL-2-dependent HTLV-1-infected SP cells were cultured in RPMI media supplemented with 20% FBS, 2 mM L-glutamine, penicillin/streptomycin and 50 U/mL IL-2. FTI-277 (Sigma, St. Louis, MO, USA), GGTI-298 (Sigma, St. Louis, MO, USA) and 9-aminoacridine (9AA) (Sigma, St. Louis, MO, USA) were solubilized in dimethyl sulfoxide (DMSO) (Sigma, St. Louis, MO, USA). During drug treatment, mock treated cells were cultured in an equal amount of DMSO (0.1%).

### 3.3. Whole Cell and Nuclear Extracts Preparation

Approximately 5 × 10^6^ cells were washed in 0.5 volumes of cold 0.01 M PBS and pelleted. For whole cell extract preparation, cells were incubated in cell lysis buffer (50 mM Tris-HCl, pH 7.4, 120 mM NaCl, 5 mM EDTA, 0.5% NP-40, 0.2 mM Na_3_VO_4_, 50 mM NaF) with 1 mM DTT, 1 mM PMSF, and protease inhibitors on ice for 20 minutes, centrifuged and the supernatant collected. For nuclear extract preparation, cells were lysed with NE-PER Nuclear and Cytoplasmic Extraction Reagents according to manufacturer’s instructions (Pierce, Rockford, IL, USA). Protein concentrations were determined by Bradford assay.

### 3.4. Immunoblot Analysis

Protein samples of 20 to 50 μg was separated by sodium dodecyl sulfate-polyacrylamide gel electrophoresis (4–12% or 12%) in MES SDS running buffer (Invitrogen, Carlsbad, CA, USA) and transferred to Immobilon-P membranes (Millipore, Billerica, MA, USA). The membranes were blocked with PBS containing 0.1% Tween and 2.5% nonfat dried milk, dried and then probed with the appropriate primary and horseradish peroxidase-conjugated secondary antibodies. Immunoreactivity was detected with SuperSignal West Pico chemiluminescent substrate (Pierce, Rockford, IL, USA). Densitometric volume analyses were performed with AlphaEase FC (Alpha Innotech, Santa Clara, CA, USA) [48]. Protein levels were normalized to actin levels from the same well. Normalized protein levels were compared with control protein level, which was set to 1, to determine relative protein levels.

### 3.5. Antibodies

IκB, p-IκB, (Cell Signaling, Danvers, MA, USA), actin (Sigma, St. Louis, MO, USA), and p53 (Calbiochem, San Diego, CA, USA), primary antibodies and horseradish peroxidase-conjugated secondary antibodies (GE Healthcare, Chalfont St. Giles, UK) were purchased. Tab172 monoclonal antibodies were used to detect the expression of Tax protein.

### 3.6. Cell Viability Assays

Cell viability assays were performed using the CellTiter-Glo luminescent cell viability assay (Promega, Madison, WI, USA) according to the manufacturer’s instructions. Briefly, 2.5 × 10^5^ cells/mL were cultured in sterile 96-well plates in the presence of GGTI-298 or DMSO in RPMI medium. Following incubation, 100 μL of CellTiter-Glo reagent was added to lyse the cells. The contents were mixed on a rocker for 2 minutes and then incubated at room temperature for 10 minutes. Luminescence was recorded in a luminometer with an integration time of 1 second per well. The luminescent signals from the GGTI-298-treated cells were normalized to the luminescent signal from cells treated with DMSO alone, which was arbitrarily set to 100%.

### 3.7. Flow Cytometry Analysis of Cell Cycle Progression

One to 2 × 10^6^ cells were treated with 10 μM GGTI-298 or DMSO for 48 hours, washed in PBS, and fixed in 5 mL 70% ethanol overnight at −20 °C. Cells were washed in PBS and stained with 0.5 mL propidium iodide staining buffer (50 μg/mL propidium iodide diluted in PBS with 0.1% Triton-X-100) for 20 minutes. Samples were acquired on a FACSCalibur flow cytometer using ModFit LT software (Verity Software House, Topsham, ME, USA) [49] and data was analyzed using FlowJo software (Tree Star, Ashland, OR, USA) [50].

### 3.8. Transfection and Luciferase Assays

Three to 4 × 10^6^ cells were transfected with 1 μg RSV-β-galactosidase and 4 μg 4×-NF-κB-Luc, PG13-Luc, or HTLV-1-LTR-Luc luciferase reporter plasmids by nucleofection with Human T-cell Nucleofector Kit/nucleofection program U-014 (Amaxa, Gaithersburg, MD, USA) according to manufacturer’s instructions. All plasmids were previously described [13]. Immediately after nucleofection, 5 × 10^5^ cells/well were distributed on 24 well-plates. Four hours after nucleofection, cells were treated with GGTI-298, 9AA or DMSO. Forty-eight hours after treatment, cells were lysed in passive lysis buffer and luciferase activity was measured using the dual luciferase reporter assay system (Promega, Madison, WI, USA) and Galactolight assay kit (Tropix, Bedford, MA, USA). Luminescence was recorded in a luminometer with an integration time of 12 seconds per sample. Luciferase activity was normalized to β-galactosidase activity from the same well. Normalized luciferase activity from cells treated with DMSO solvent alone was set to 1 and luciferase values from each drug concentration were determined relative to this value.

### 3.9. Real-Time PCR

For real-time PCR analysis, 1 × 10^6^ cells were treated with 10 μM GGTI-298 or DMSO for 48 hours. Cells were washed in PBS and total RNA was extracted from all samples with RNeasy Plus (Qiagen, Valencia, CA, USA) and retrotranscribed with Quantitect Reverse Transcription kit (Qiagen, Valencia, CA, USA), according to manufacturer’s instructions. The cDNA quantification for *18s*, *IL6*, *iNOS*, *MDM*, *PIG3* and *tax/rex* was performed by real-time PCR using an ABI 7000. Reactions were performed using a SYBR FAST PCR mix (Kapa Biosystems, Woburn, MA, USA), following the manufacturer’s instructions. Primer sequences were designed to specifically amplify:

*18s*, 5′-GCCCGAAGCGTTTACTTTGA-3′ (forward) and
5′-TCCATTATTCCTAGCTGCGGTATC-3′ (reverse);
*IL6*, 5′-GGTACATCCTCGACGGCATC-3′ (forward) and
5′-CCAGTGCCTCTTTGCTGCTT-3′ (reverse);
*iNOS*, 5′-CAAGGCATCCTGGAGCGAGT-3′ (forward) and
5′-GTAGGTGAGGGCCTGGCTGA-3′ (reverse);
*MDM*, 5′-GGTTGACTCAGCTTTTCCTCTTG-3′ (forward) and
5′-GGAAAATGCATGGTTTAAATAGCC-3′ (reverse); *PIG3*, 5′-CACTCCCAACGGCTCCTTT-3′ (forward) and 5′-GCCCATCTTGAGCATGGGTG-3′ (reverse); *tax/rex* primers were previously described [51]. Results were expressed as ΔΔCt, where Ct is the cycle threshold, and presented as ratios between the target gene and the *18s* housekeeping RNA. Normalized mRNA levels from cells treated with DMSO solvent alone was set to 1 and mRNA levels from drug-treated cells were determined relative to this value.

### 3.10. Electrophoretic Mobility Shift Assays (EMSAs)

The sequence used for NF-κB consensus wild-type oligonucleotide was 5′-AGTTGAGGGGACTTTCCCAGGC-3′, while the NF-κB mutant oligonucleotide was 5′-AGTTGAGGCGACTTTCCCAGGC-3′. Double-stranded NF-κB consensus wild-type oligonucleotide was labeled with biotin-11-dUTP (Pierce, Rockford, IL, USA). EMSA reactions included 10× EMSA buffer (100 mM Tris pH7.5, 500 mM KCl, 10 mM DTT, 2.5% glycerol, 5 mM MgCl, 50 ng poly(dI/dC), 2.5 μg nuclear extract and approximately 20 fmol labeled probe in a 20 μL total reaction volume. For competition assays, approximately 200-fold molar excess of unlabeled oligonucleotide was added to the reaction. Reactions were incubate6+d at room temperature for 20 minutes. Complexes were resolved on a 5% nondenaturing polyacrylamide gel in 0.5× TBE. Following electrophoresis, binding reactions were transferred to a Biodyne B membrane (Pierce, Rockford, IL, USA) and complexes were detected by streptavidin-horseradish peroxidase chemiluminescent substrate.

### 3.11. Adenovirus Infection of Cells

An adenovirus-Tax (Ad-Tax) construct provided by M. Yoshida and an adenovirus-green fluorescent protein (Ad-GFP) construct (Q-Biogene, Montréal, Canada) were used to infect HTLV-1-transformed C8166 cells (1 × 10^6^ cells) in RPMI media not supplemented with serum. The adenovirus constructs were previously described [52]. At three hours post-infection, cells were washed and resuspended in RPMI media supplemented with 10% FBS, 2 mM L-glutamine and penicillin/streptomycin. The infected cells were incubated for 24 hours for GFP and Tax expression and then treated with 10 μM GGTI-298 for an additional 24 hours.

### 3.12. Statistical Analysis

Data were analyzed by Student’s two-sample equal variance *t* test with a *p* value of <0.05 considered significant.

## 4. Conclusions

In conclusion, we have demonstrated that treatment of HTLV-1-transformed cells with GGTI-298, a prenylation inhibitor that inhibits geranylgeranylation, decreased cell viability and induced G_2_/M phase accumulation. The effects of GGTI-298 observed in HTLV-1-transformed cells suggest an important role for geranylgeranylated proteins in cell survival and cell cycle progression. Continued study and characterization of geranylgeranylated proteins, such as Rho family GTPases, will increase our understanding of the mechanisms that regulate transformation of HTLV-1-infected cells.
